# Retention of HIV exposed infants in care at Arua regional referral hospital, Uganda: a retrospective cohort study

**DOI:** 10.1186/s12889-019-6754-9

**Published:** 2019-04-25

**Authors:** Pontius Apangu, Jonathan Izudi, Francis Bajunirwe, Edgar Mulogo, Vincent Batwala

**Affiliations:** 1 Department of Community Health, Faculty of Medicine, Mbarara University of Science and Technology, Box 1410, Mbarara, Uganda; 2 Institute of Public Health and Management, Clarke International University, Box 7782, Kampala, Uganda; 3 Directorate of Research and Graduate Training, Mbarara University of Science and Technology, Box 1410, Mbarara, Uganda

**Keywords:** Retention, HIV exposed infants, Early infant diagnosis of HIV, Uganda

## Abstract

**Background:**

Retention of HIV Exposed Infants (HEIs) in care ensures adequate care. Data on retention of HEIs at large referral hospitals in Uganda is limited. We investigated the retention level of HEIs and associated factors.

**Methods:**

We conducted a retrospective cohort study on 352 HEIs in care (January 2014 and April 2015) at Arua Regional Referral Hospital, North-western Uganda. Electronic medical data were retrieved and analyzed with Stata. Chi-square, Fisher’s exact, and Students t-tests were used for bivariate analysis. Logistic regression was performed to determine factors independently associated with retention.

**Results:**

236 (67.0%) HEIs were delivered in a health facility and 306 (86.9%) received Nevirapine prophylaxis from birth until 6-weeks. Of mothers, 270 (76.7%) were 25–46 years, 202 (57.4%) attended antenatal care (ANC) at recent pregnancy, and 328 (93.2%) were on life-long anti-retroviral therapy. At 18-months, 277 (78.7%) HEIs were retained in care. Maternal age (25–46 years) (Adjusted Odds Ratio (AOR), 2.32; 95% CI, 1.32–4.06), ANC attendance during recent pregnancy (AOR, 2.01; 95% CI, 1.19–4.3.41) and Nevirapine prophylaxis initiation from birth until 6-weeks (AOR, 3.07; 95% CI, 1.50–6.26) were associated with retention.

**Conclusion:**

Retention was suboptimal. Older maternal age, ANC visits at last pregnancy, and timely NVP initiation increased retention.

## Background

Despite the nationwide implementation of Elimination of Mother to Child transmission of Human Immunodeficiency Virus (HIV), abbreviated as EMTCT, and Early Infant Diagnosis of HIV (EID) policies, retention of HIV Exposed Infants (HEIs) in care remains suboptimal [1]. Under EMTCT strategy, HIV positive pregnant and breastfeeding women are enrolled into EID continuum of care at the Mother-baby Care Point, within the Maternal and Child Health (MCH) clinic. HIV positive breastfeeding mothers and HEIs are paired, then followed for 18 months. During the follow-up, Dry Blood Spot (DBS) samples are collected from HEIs between six and eight weeks and at 6-weeks after cessation of breastfeeding for HIV testing using Deoxyribonucleic Acid Polymerase Chain Reaction (DNA-PCR), and a final HIV antibody test is performed at 18-months [2]. Infants who test positive for HIV are started on life-long ART irrespective of age, immunological status, and World Health Organization (WHO) clinical stage. However, infants who test negative for HIV at 18-months are discharged from the EID program as HIV uninfected.

Along the EID continuum of care, data shows that retention of HEIs declines [3–7]. In Malawi for instance, 48% of HEIs were lost by 24-months [8], and approximately 58% were lost by the end of 30-months [9]. In a multicentre study conducted across four African countries (Mozambique, Rwanda, Kenya, and Tanzania), substantial proportion of HIV positive infants started on Anti-retroviral Therapy (ART) were lost [6]. Loss of HEIs is one major factor that accounts for low retention along the EID continuum of care, besides several others [10]. Epidemiological studies indicate that long hours spent travelling to a health facility and associated costs [11], socio-economic factors [12–14], maternal factors like inadequate antenatal care (ANC) attendance [15, 16], age [17], marital status [4], inadequate understanding of the benefits of follow-up of HEIs and use of ART [18, 19] are associated with low retention of HEIs in care.

Equally, health services-related factors like place of delivery [14, 19], long health facility waiting time, negative attitudes of healthcare providers, inadequate counselling of mothers, inappropriate location of EID clinics within health facilities, lack of patient privacy and confidentiality of patient information [20–22], are associated with low retention of HEIs in care.

In 2014, Arua Regional Referral Hospital (ARRH), North-western Uganda commenced the implementation of EID policy under the EMTCT strategy. However, the level of retention of HEIs at the end of 18-months of EID continuum of care and associated factors has never been evaluated. In this study, we investigated the level of retention of HEIs at the end of 18-months of EID continuum of care and the associated maternal and infant factors at ARRH, North-western Uganda. The result of this evaluation will inform practice and programing for improved EID service delivery.

## Methods

### Study design and reporting

The reporting of the study results followed the Strengthening of the Reporting of Observational Studies in Epidemiology (STROBE) guidelines [23, 24]. We used a retrospective cohort study to determine the level of retention of HEIs at the end of 18-months of EID continuum of care and associated factors at ARRH in North-western Uganda. This study design was appropriate because existing data in which both exposure and outcome had occurred were abstracted, organized according to exposure and outcome, and associations between exposures and outcome established [25]**.**

### Study setting, study population, sampling, and sample size

This study was conducted at ARRH, one of the 13 Regional Referral Hospitals in Uganda.

ARRH offers both specialized and general health services as well as supervisory role to four District Government Hospitals, four Private-Not-for-Profit General Hospitals, and 11 County-level Health Centres in the West Nile region. ARRH was purposively selected because it is the first hospital in the West Nile region to commence the provision of comprehensive HIV/AIDS care. From 2012 to date, available data indicates that ARRH has cumulatively enrolled over 2000 HEIs in care. Of HEIs enrolled in care, 352 were enrolled between January 2014 and April 2015 (the study period). In this study, all the 352 HEIs were retrospectively sampled by census and followed from the time of entry into the EID program until 18-months of age. In terms of clinical care, every month, each HEI received clinical review and care at the Mother Baby Care Point (MBCP).

### Data collection

We reviewed and extracted medical data from the HIV clinic electronic database for mother-baby pairs enrolled in care between January 2014 and April 2015, in October 2016.

In particular, we used the EID register, clinical charts, and dispatch books to authenticate data whenever a disparity was noted in the data abstracted from the electronic database.

### Study variables

We extracted data on maternal and infant variables. Maternal variables included age at enrolment into EID care in absolute years (later categorized as below 25-years, or greater than or equals 25-years), baseline CD4 counts in cells/ul, baseline viral load in copies/ml, ART use (ART naive, ART use during labor and delivery only, or life-long ART), and ANC use during recent pregnancy (never used, or ever used). Infant variables included age in months, feeding option in the first 6-months of life (mixed feeding, exclusive breastfeeding, or replacement feeding), mode of delivery (spontaneous vaginal delivery, or caesarean section), place of delivery (health facility or home), and time of NVP (Nevirapine) prophylaxis initiation (never, after 6-weeks, and between birth and 6-weeks).

The dependent variable was retention of HEIs at the end of 18-months of EID continuum of care. Retention was measured as a binary outcome (no or yes). HEIs who remained HIV-negative along the 18-months of EID continuum of care (at 6-weeks, at 1-year, or at 18-months), or who tested HIV positive and were started on ART, or who were transferred out of ARRH to other EID providing health facilities were considered retained. Conversely, HEIs who died, or who tested HIV positive but were not started on ART, or who got lost along the EID continuum of care were considered non-retained.

### Statistical analysis

Retrieved data were cleaned and exported to Stata version 12 (StataCorp, College Station, TX, USA) for univariate, bivariate and multivariate analysis at 5% significance level. In univariate analysis, measures of central tendency such as mean with standard deviation, or median with interquartile ranges were calculated for numerical variables, and frequencies and percentages were computed for categorical variables.

We computed retention inform of a percentage. The numerator was the sum of the number of HEIs who remained HIV-negative along the 18-months EID continuum of care (at 6-weeks, at 1-year, or at 18-months), HEIs who tested HIV positive and were started on ART, and HEIs who were transferred out of ARRH to other EID providing health facilities. The denominator was the sample size.

To establish the relationship between categorical variables and retention in bivariate analysis, the Chi-square test was employed for larger cell counts (equal and above five), while the Fisher’s exact test was used for smaller cell counts (less than five). Conversely, to determine the relationship between numerical independent variables and retention, we used the Student’s *t*-test. Associations with probability value (*P*-value) less than 5% at bivariate analysis were considered statistically significant for multivariate analysis.

In multivariate analysis, we first performed unadjusted binary logistic regression analysis to determine the strength of association between statistically significant variables at bivariate analysis. We reported the results in unadjusted odds ratio (UOR) and 95% confidence interval (CI). Second, we conducted a multivariable backward stepwise binary logistic regression analysis for all statistically significant associations at unadjusted analysis to determine factors independently associated with retention of HEIs in care. We reported the results in adjusted odds ratio (AOR) and 95% CI. Prior to multivariable logistic regression analysis, we performed multicollinearity test and excluded collinear variables (variables with Variance Inflation Factor (VIF) greater than 10).

We did not report *P*-values associated with odds ratios because they are less-informative and do not provide information on clinical importance [26]. In comparison, the CI is appropriate for reporting the precision of effect measures and establishing statistical significance [26]. Since the CI is more informative [27], it is presently preferred in reporting effect measures [28].

### Ethical approval

This study was approved by the Faculty of Medicine Research Ethics Committee (FREC) of Mbarara University of Science and Technology (MUST) and the MUST Research Ethics Committee (MUST-REC) which was established in February 2002 (Reference Number: DMS 6, dated June 20, 2017). MUST-REC was accredited by the Uganda National Council for Science and Technology (UNCST) in 2011, and is registered with the Federal Wide Assurance in the United States (registration number: FWA 00007740) since 2012. We did not obtained written or verbal informed consent from study participants since secondary data in existing medical records were extracted for analysis. The need for informed consent was waived by the ethics committee. Additional approval was obtained from Arua District Local Government Health Office.

## Results

### Characteristics of infants and their mothers

236 (67.0%) of the HEIs were delivered in a health facility and 292 (83.0%) were delivered by spontaneous vaginal delivery. Some 306 (86.9%) of the HEIs received Nevirapine prophylaxis from birth up to 6-weeks. 270 (76.7%) of the mothers were in the age group of 25–46 years, 202 (57.4%) attended ANC visits during recent pregnancy, 328 (93.2%) were on life-long ART, 264 (75.0%) were in WHO clinical stage one at baseline, and 208 (59.1%) had over 500 cells/ml of CD4 count at baseline (Table 1).

**Table 1:** Characteristics of infants and their mothers

Characteristics	No. (*n* = 352)	Percentage (% = 100)
Birth place		
Home	116	33.0
Facility	236	67.0
Mode of delivery		
Caesarea section	60	17.0
Spontaneous Vaginal delivery	292	83.0
Time of NVP initiation		
None	42	12.0
From birth to 6-weeks	306	86.9
After 6-weeks of birth	4	1.0
Maternal age at enrolment into EMTCT in years		
15–24	82	23.3
25–46	270	76.7
Infant Feeding option in the first 6-months		
Replacement feeding	8	2.2
Mixed feeding	56	16.0
Exclusive Breast feeding	288	81.8
ANC use during recent pregnancy		
No	150	42.6
Yes	202	57.4
Maternal ART use		
During ANC only	10	2.8
During ANC and labour only	14	4.0
Life-long ART	328	93.2
Baseline WHO Clinical staging		
I	264	75.0
II	52	14.8
III/IV	36	10.2
Baseline maternal CD4 count (cells/ml)		
Less than 350	91	25.9
350–500	53	15.0
More than 500	208	59.1

### Retention of HEIs along the 18-months EID continuum of care

**Fig. 1:**
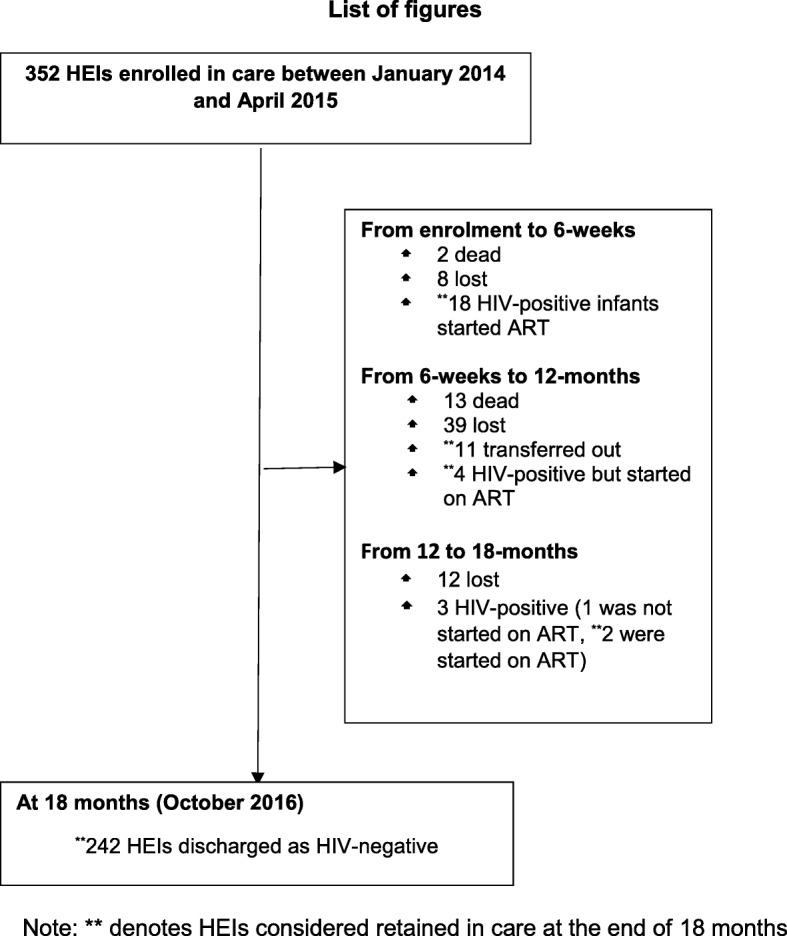
Retention of HEIs along the EID cascade, ARRH (January 2014 to April 2015)

In this study, 352 HEIs were enrolled in EID care between January 2014 and April 2015. Between enrolment and 6-weeks, two HEIs died, eight got lost and 18 turned HIV-positive on the first DNA-PCR test but were all started on ART. Between 6-weeks and 12-months, 13 HEIs died, 39 got lost, 11 were transferred to other health facilities, and four turned HIV-positive on the second DNA-PCR test but were all started on ART. Between 12 and 18-months, 12 HEIs got lost, three HEIs turned HIV-positive at 18-months on HIV antibody testing but only two were started on ART. At the end of 18-months, 242 HEIs remained HIV-negative (Fig. 1).

Overall, HEIs retained in care included; 1) Between enrolment and 6 weeks, 18 HEIs who were started on ART after turning HIV positive; 2) Between 6 weeks and 12 months, 11 HEIs who were transferred out of the EID program to other health facilities and another four HEIs who were started on ART after turning HIV positive; 3) Between 12 months to 18 months, two additional HEIs who were started on ART after turning HIV positive; and 4) At 18 months, the 242 HEIs who remained HIV negative. Accordingly, 277 (78.7%) HEIs were retained in care at the end of 18-months EID continuum of care.

### Bivariate analysis of factors associated with retention

Health facility delivery was associated with increased retention compared to home deliveries. A total of 82 (70.7%) HEIs delivered at home compared to 195 (82.6%) delivered in a health facility were retained in care (*p* = 0.01). Some 37 (61.7%) HEIs delivered by Caesarean-section compared to 240 (82.2%) by spontaneous vaginal delivery were retained in care (*p* < 0.001).

Again 22 (52.4%) HEIs who never received NVP prophylaxis after birth, 252 (82.3%) who received NVP prophylaxis from birth until 6-weeks, and three (75.0) who received NVP prophylaxis after 6-weeks were retained in care (*p* < 0.001). 54 (65.9%) HEIs retained in care were from mothers aged 15–24 years while 223 (82.6%) were from mothers aged 25–46 years (*p* = 0.001).

Five (62.5%) HEIs who were fed by replacement, 29 (51.8%) who were mixed fed, and 243 (84.4%) who were exclusive breastfed were retained in care (*p* < 0.001). 170 (84.2%) HEIs retained in care were from mothers who never used ANC in recent pregnancy compared to 107 (71.3%) from mothers who used ANC (*p* = 0.004).

Five (50.0%) HEIs retained in care were from mothers who received ART during ANC only, six (42.9%) were from mothers who received ART during labor and delivery only, and 62 (18.9%) were from mothers who were on life-long ART (*p* < 0.001) (Table 2).

**Table 2:** Multivariate logistic regression analysis of factors associated with retention of HEIs in care, Arua Regional Referral Hospital

Characteristics	Retained in care at 18-months?		Univariable logistic regression analysis (UOR, 95% CI)	Multivariable logistic regression analysis (AOR, 95% CI)
	No	Yes	UOR (95%CI)	AOR (95%CI)
Birth place				
Home	34 (29.3)	82 (70.7)	1	1
Facility	41 (17.4)	195 (82.6)	1.97^*^ (1.17–3.33)	1.62 (0.94–2.79)
^a^Mode of delivery				
Caesarea section	23 (38.3)	37 (61.7)	1	
Normal	52 (17.8)	240 (82.2	2.87^***^ (1.57–5.23)	
Time of NVP initiation				
None	20 (47.6)	22 (52.4)	1	1
From birth to 6-weeks	54 (17.7)	252 (82.3)	4.24^***^ (2.16–8.32)	3.07^**^ (1.50–6.26)
After 6-weeks of birth	1 (25.0)	3 (75.0)	2.73 (0.26–28.39)	3.06 (0.23–34.03)
Maternal age at enrolment into EMTCT in years				
15–24	28 (34.1)	54 (65.9)	1	1
25–46	47 (17.4	223 (82.6)	2.46^**^ (1.41–4.28)	2.32^**^ (1.32–4.06)
Infant Feeding option in the first 6-months				
Replacement feeding	3 (37.5)	5 (62.5)	1	
Mixed feeding	27 (48.2)	29 (51.8)	0.64 (0.14–2.96)	
Exclusive Breast feeding	45 (15.6)	243 (84.4)	3.24 (0.75–41.04)	
ANC use during recent pregnancy				
No	32 (15.8)	170 (84.2)	1	1
Yes	43 (28.7)	107 (71.3)	2.13^**^ (1.27–3.58)	2.02^**^ (1.19–3.41)
^a^Maternal ARV use				
During ANC only	5 (50.0)	5 (50.0)	1	
During ANC and labour only	8 (57.1)	6 (42.9)	0.75 (0.15–3.83)	
For life	62 (18.9)	266 (81.1)	4.29^*^ (1.20–15.28)	

Exponentiated coefficients; 95% confidence intervals in brackets

^*^ *p* < 0.05, ^**^ *p* < 0.01, ^***^ *p* < 0.001

In Table 2, percentages were calculated as row percentage (n/N); *ANC* Antenatal care, *AOR* Adjusted Odds Ratio, *UOR* Unadjusted Odds Ratio; ^a^: Collinear variables with variance inflation factor greater than 10

### Univariable logistic regression analysis results

Health facility delivery (UOR, 1.97; 95% CI, 1.17–3.33), spontaneous vaginal delivery (UOR, 2.87; 95% CI, 1.57–5.23), receipt of NVP prophylaxis from birth until 6-weeks (UOR, 4.24; 95% CI, 2.16–8.32) and after 6-weeks (UOR, 2.73; 95% CI, 0.26–28.39), maternal age greater than or equal to 25-years at enrolment into EMTCT (UOR, 2.46; 95% CI, 1.41–4.28), excusive breastfeeding of HEIs during the first 6-months (UOR, 3.24; 95% CI, 0.75–41.04), ANC use during recent pregnancy (UOR, 2.13; 95% CI, 1.27–3.58), and maternal use of life-long ART (UOR, 4.29; 95% CI: 1.20–15.28) were associated with higher odds of retention of HEIs in care. However, retention reduced with mixed feeding of HEIs (UOR, 0.64; 95% CI, 0.14–2.96) and maternal use of ART during labor and delivery only (UOR, 0.75; 95% CI, 0.15–3.83) (Table 2).

### Multivariable logistic regression analysis results

Prior to multivariate analysis, we found maternal ART use and mode of delivery were collinear. Accordingly, we excluded them from the multivariable logistic regression analysis. After adjustment, NVP initiation from birth until 6-weeks (AOR, 3.07; 95% CI, 1.50–6.26), maternal age of 25–46 years at enrolment into EMTCT (AOR, 2.32; 95% CI, 1.32–4.06), and maternal ANC attendance during recent pregnancy (AOR, 2.01; 95% CI, 1.19–3.41) were statistically significantly associated with retention of HEIs in care (Table 2).

## Discussion

This study investigated the level of retention of HEIs along the 18-months of EID continuum of care and associated factors at ARRH. We found 78.7% of HEIs were retained in care, which is suboptimal compared to the required 90% level of retention for EMTCT by 2020 [29]. The retention level is also significantly higher than the national level of 56% [30]. The present level of retention of HEIs indicates that ARRH is on a positive trend to achieving the ambitious global target of retaining 90% or more HEIs in care. However, to improve retention, ARRH needs to take deliberate steps like using appointment books to fast-track mother-baby pairs during the 18 months of EID continuum of care, mobile phone text messaging to remind mothers of their clinic appointments, mobile phone calls to fast-track lost mother-baby pairs, and home visits for lost mother-baby pairs [31]. Previously in Uganda, these interventions led to 60% retention of HIV positive pregnant and lactating mothers, and 75% retention of HIV positive children in care at the end of 1-year [31]. Retention of HEIs is critical because non-retained HEIs have high risk of morbidity and mortality particularly, when they turn HIV positive and are not started on ART [32, 33]. The current level of retention of HEIs is comparable to an earlier study in Rwanda that found 74% level of retention at the end of 18-months [4].

In general, retention of HEIs appears to be a major problem across HIV programs in Africa. In a previous multi-center study in Senegal, Uganda, Cambodia, and Namibia [34], the levels of retention were 22, 37, 38, and 70%, respectively. Only Cameroon recorded a high level of retention of HEIs at 83.9% [35], much higher than in this study. The difference in retention levels can be explained by differences in follow-up period. The present study retrospectively followed HEIs for 18-months while the Cameroonian study followed HEIs for only 7-months. We found retention of HEIs reduced along the EID continuum of care, which is consistent with levels of retention of 73.8% at 6-weeks and 25.8% at 1-year in a Kenyan study [36].

Mothers who were 25-years old or more at enrolment into EMTCT had higher retention of HEIs compared to mothers who were less than 25-years old. Studies in Cameroon [37] and Kenya [17] have reported that older maternal age is associated with retention of HEIs in care. The plausible explanation for this difference could be attributed to the overall time spent in HIV care. The older mothers might have spent more time in EID care than younger mothers, and are therefore better experienced in caring for HEIs than younger mothers. However, regardless of the age differences, all mother-baby pairs must be retained in care. This will prevent maternal and infant HIV-related morbidity and mortality, and ultimately achieve the Sustainable Development Goal of health for all. Our results suggests the need for conducting an additional research in evaluating the effect of maternal age differences on retention of HEIs in care at ARRH.

The level of retention of HEIs is high among mothers who attended ANC visits during recent pregnancy compared to those who did not. This result demonstrates the importance of ANC in improving maternal and newborn health and survival. First, in Uganda, ANC is an important gateway into accessing EMTCT interventions [30]. For pregnant women, ANC offers a golden opportunity for HIV diagnosis, post-test HIV counselling, and health education on positive living. Essentially, ANC provides mothers with sufficient health information resulting into raised self-efficacy to effectively control the determinants of health. The study result reinforces the importance of encouraging and supporting all pregnant women in early initiation and completion of all required ANC visits. Importantly, the provision of high quality ANC to pregnant women is critical in reducing missed opportunities. In Ethiopia [19, 38] and Malawi [39], studies indicates that mothers who attend ANC visits have superior retention of HEIs in care compared to those who do not attend ANC visits.

HEIs who were started on Nevirapine (NVP) prophylaxis from birth until 6-weeks were more retained in care compared to those who were never started on NVP prophylaxis. Currently, we did not find published literature to support this result. Whereas retention should not be explained on the basis of NVP prophylaxis, there is a possibility that receipt of routine health education messages, ART adherence counselling, and psychosocial support accompanying monthly NVP prophylaxis refills could have played an immense role in enhancing retention. In practice, healthcare providers should support mother-baby pairs to receive the entire EID package. This will enable mother-infant pairs to remain in care until 18-months, and for as long as required.

### Study strengths and limitations

Our study underscored important maternal and infant factors associated with retention of HEIs along the 18-months of EID continuum of care. This study has several strengths. To our knowledge, it is the first study at ARRH. We studied retention of HEIs over a relatively longer length of time (18-months) and all HEIs considered retained were still active in care. Second, none of the HIV positive infants started on ART had defaulted from the EID program at the time of this study. Likewise, several pitfalls should be taken into account in interpreting the results. First, we used secondary data initially collected for clinical care rather than research purposes. Consequently, the numbers of variables were limited for studying retention comprehensively. Second, the exclusion of HEIs with missing data also reduced the sample size that otherwise would have improved the precision of the results. Third, the absence of qualitative data to explain the quantitative results is another drawback worthy of mentioning. Fourth, our outcome was large, approximately 79%. Under such situations, the odds ratios overestimate the degree of association between the exposure and the outcome. Ideally, prevalence rate ratios computed using Modified Poisson regression analysis is recommended [40–43].

## Conclusions

This study indicates that approximately 79% of HEIs were retained in care at the end of 18 months in ARRH, which is suboptimal compared to the required 90% level of retention for EMTCT. Older maternal age (25-years and over at enrolment into EMCT), ANC attendance during recent pregnancy, and NVP prophylaxis initiation from birth until 6-weeks of age were associated with increased retention of HEIs in care. To enhance retention of HEIs in care, all HIV positive mothers must receive correct and adequate HIV counseling and psychosocial support during ANC visits.

## Abbreviations

ANC: Antenatal Care
AOR: Adjusted Odds Ratio
ARRH: Arua Regional Referral Hospital
ART: Anti-Retroviral Therapy
EID: Early Infant Diagnosis of HIV
EMTCT: Elimination of Mother to Child Transmission of HIV
HEIs: HIV Exposed Infants
HIV: Human Immunodeficiency Virus
UOR: Unadjusted Odds Ratio
